# Supplementary material for: Synergistic Membrane Disturbance Improves the Antibacterial Performance of Polymyxin B
Source: Polymers (Basel). 2022 Oct 14;14(20):4316. doi: 10.3390/polym14204316 (PMC9611124; doi:10.3390/polym14204316)
Supplement: Supplementary file 1 [file polymers-14-04316-s001.zip › polymers-1938456-supplementary.pdf]

Supporting Information for

Synergistic Membrane Disturbance Improves the Antibacterial Performance of Polymyxin B

[1] Center for Soft Condensed Matter Physics and Interdisciplinary Research, School of Physical Science and Technology, Soochow University, Suzhou 215006, China

[2] Songshan Lake Materials Laboratory, Dongguan 523808, China

[3] School of Optical and Electronic Information, Suzhou City University, Suzhou 215104, Jiangsu, China

* Correspondence: yangkai@suda.edu.cn (K.Y.); yuanbing@sslab.org.cn (B.Y.)

† These authors contributed equally to this work

Supplementary images and tables.

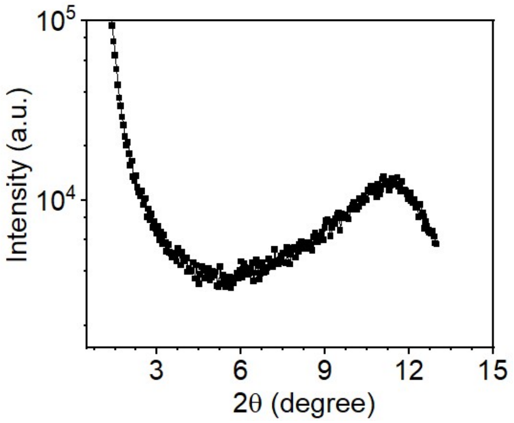

Figure S1. Powder XRD pattern of the GO film deposited on the surface of a silicon wafer. The prominent peak at ~11.5° represents the lamellar structure of GO sheets, corresponding to an inter-layer spacing of ~7.7 Å.

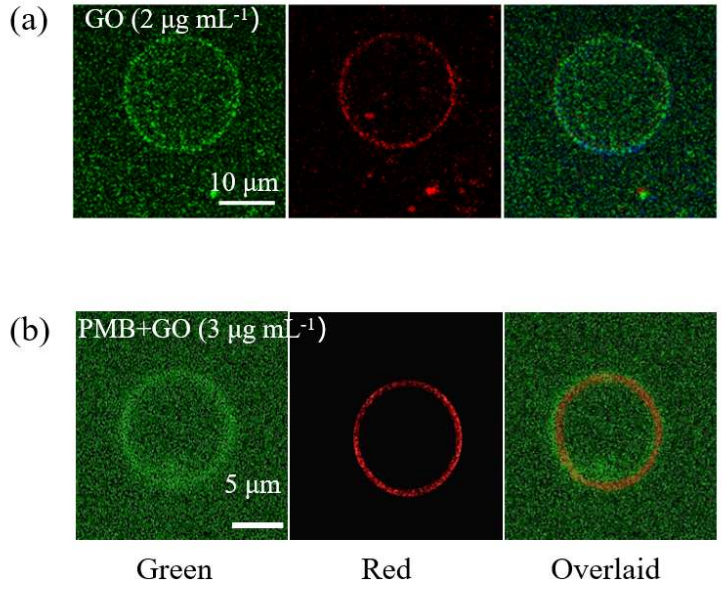

Figure S2. GUV leakage due to the addition of (a) free GO or (b) PMB+GO mixture. A bright green circle (i.e., calcein) is found overlaid with lipids after GO actions. (a) DOPC GUV with GO at 2.0 μg mL^{-1}. (b) PC/PG/Chol GUV (3:1:1 by mol) with PMB+GO mixture at 3.0 μg mL^{-1} (with respect to peptide; PMB:GO = 8:1 by wt, as stated in the main text). The images show representative vesicles 30 min after the addition of agents.

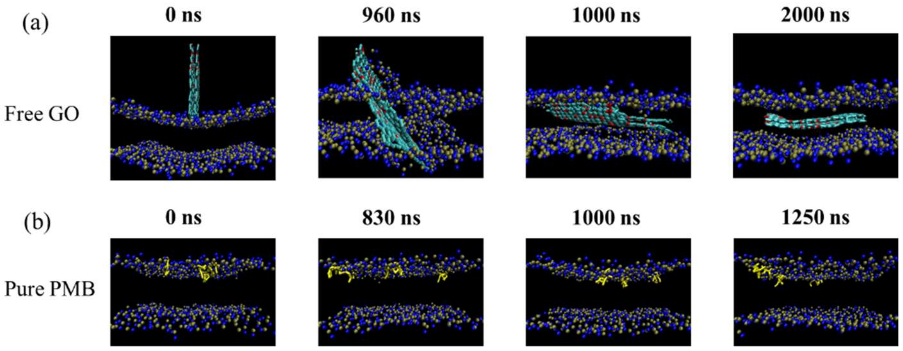

Figure S3. Simulation snapshots of the membrane interactions of (a) free GO or (b) pure PMB. The GO sheet in (a) has a size of 7.2×6.3 nm. In (b), three PMBs are added. The membrane is composed of 512 DOPC lipids. Blue and grey beads in snapshots: phospholipid headgroups; yellow rod: PMB; light blue sheet with red beads: GO with hydrophilic groups. Lipid tails are not shown for clarity.

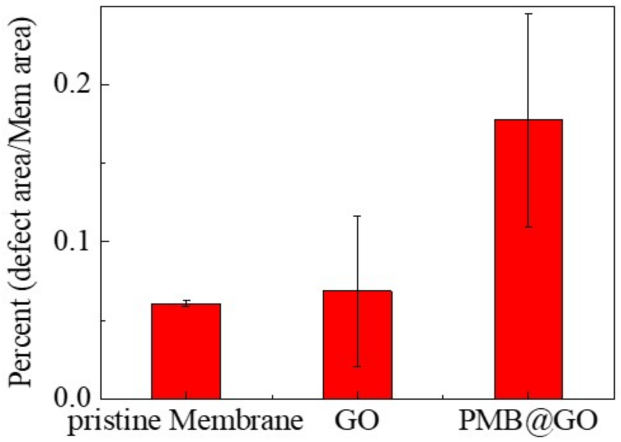

Figure S4. Distribution of the membrane defects due to agent addition. For comparison, that of the pristine membrane is also shown.

Table S1. Hemolytic activity tests of free PMB and PMB@GO nanocomposite. The red blood cells were pretreated with PBS (as negative control), ultrapure water (as positive control), pure PMB, or PMB@GO, respectively, for 4 h before centrifugation and determination. The table shows the results of only one experiment.

**Hemolytic activity**	**Experimental Group （μg/ml）**							**Positive Control**	**Negative Control**
	0.25	0.5	1	2	4	8	16		
**PMB**	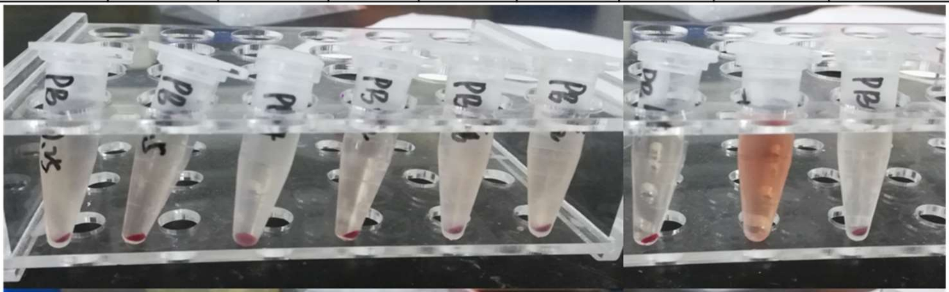								
**GO-PMB**	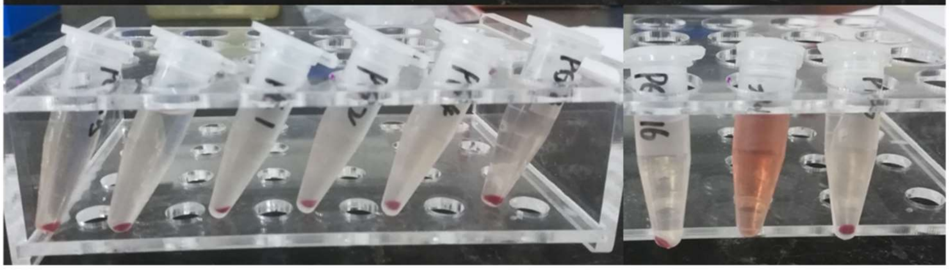								

*Precipitation indicates intact red blood cells.
